# Long-term wound morbidity and hernia recurrence in diabetic patients with HbA1c ≥ 8.5% undergoing open transversus abdominis release: a descriptive longitudinal follow-up of a single-center cohort

**DOI:** 10.1007/s10029-026-03758-9

**Published:** 2026-06-15

**Authors:** Fahim Kanani, Narmin Zoabi, Benjamin T Miller, Lucas RA Beffa, Clayton C Petro, Ajita S Prabhu, Michael J Rosen, Nir Messer

**Affiliations:** 1 Department of Surgery, Tel Aviv Sourasky Medical Center and Gray Faculty of Medicine, Tel -Aviv University, Tel Aviv, Israel; 2 Cleveland Clinic Center for Abdominal Core Health, Department of General Surgery, Cleveland Clinic Foundation, Cleveland, OH USA; 3 Department of Gastroenterology, Gray Faculty of Medicine, Sheba Medical Center, Ramat Gan, Israel; 4 Department of Surgery, Gray Faculty of Medicine, Wolfson Medical Center, Holon, Israel; 5 Division of GI Surgery, Northwestern University and Feinberg School of Medicine, Chicago, IL USA

**Keywords:** Ventral hernia repair, Abdominal wall reconstruction, Transversus abdominis release, Diabetes mellitus, Glycemic control, HbA1c, Surgical site infection, Surgical site occurrence, Hernia recurrence, Preoperative optimization

## Abstract

**Background:**

Preoperative glycemic control, as measured by HbA1c, is widely used in risk stratification for diabetic patients undergoing abdominal wall reconstruction (AWR). In a prior analysis, our group demonstrated that among diabetic patients undergoing open ventral hernia repair (VHR) with transversus abdominis release (TAR), preoperative HbA1c levels did not correlate with short-term wound morbidity. This study evaluates long-term outcomes in patients with poor preoperative glycemic control (HbA1c ≥ 8.5%) undergoing open AWR with TAR.

**Methods:**

Adult diabetic patients with HbA1c ≥ 8.5% who underwent open, elective, clean VHR with concurrent TAR and permanent synthetic mesh at the Cleveland Clinic Center for Abdominal Core Health between January 2014 and January 2024 were identified from the Abdominal Core Health Quality Collaborative (ACHQC) and followed for a minimum of 12 months. Assessed outcomes included wound morbidity, mesh-related complications, and hernia recurrence. Threshold analysis using ROC methodology and logistic spline regression was performed to evaluate the discriminative capacity of HbA1c within the poor glycemic control range.

**Results:**

Of 48 patients with HbA1c ≥ 8.5%, 46 (95.8%) completed long-term follow-up at a median of 22.3 months. Long-term SSOPI and partial mesh removal rates were 4.3% and 2.2%, respectively. Pragmatic hernia recurrence was observed in 4 patients (8.7%), all of whom had experienced early wound morbidity (4/4); three of the four had also undergone partial mesh excision or removal (3/4). No recurrences were documented in patients with uncomplicated postoperative courses. Threshold analysis yielded an AUC of 0.37, indicating no meaningful discriminative threshold within the observed HbA1c range.

**Conclusion:**

Among diabetic patients with HbA1c ≥ 8.5% undergoing open AWR with TAR at a high-volume center, long-term wound and recurrence rates were within the range reported for general TAR cohorts. These descriptive findings, while limited by sample size and the absence of an internal comparator, support the hypothesis that preoperative HbA1c may have limited stand-alone discriminative capacity in this setting and warrant confirmation in adequately powered comparative analyses, including the longitudinal multi-stratum cohort currently ongoing at our institution.

## Introduction

Preoperative optimization is a fundamental principle of elective abdominal wall reconstruction (AWR) aimed at enhancing patient safety and improving surgical outcomes [1, 2]. Diabetes mellitus (DM) is among the most well-established risk factors, consistently associated with increased postoperative wound morbidity, which may, in turn, predispose to mesh-related complications and elevate the risk of hernia recurrence [3].

Glycemic control (GC), as measured by glycated hemoglobin (HbA1c), remains a principal focus in risk stratification for diabetic patients undergoing AWR surgery [4]. HbA1c serves as an integrated marker of chronic glycemic status and long-term diabetic complications and has been widely employed as a surrogate predictor of postoperative diabetic complications [5, 6] However, much of the existing evidence originates from heterogeneous surgical populations and administrative datasets encompassing varied surgical disciplines, techniques, and patient characteristics [7]. These limitations may yield statistically significant associations with limited clinical relevance. Furthermore, there remains a paucity of high-quality, specific evidence evaluating the association between preoperative glycemic status and postoperative outcomes in AWR [3]. In a recent analysis by our group, we examined short-term wound and overall outcomes in diabetic and non-diabetic patients undergoing open AWR surgery with transversus abdominis release (TAR) procedure [3]. Our findings reaffirmed DM as an independent marker of increased wound-morbidity and overall complications. However, when diabetic patients were stratified according to preoperative HbA1c levels, no significant differences were observed in short-term outcomes, aside from a non-significant trend toward increased noninfectious seroma formation.

These findings have prompted scrutiny, particularly in light of the 30-day postoperative window used for outcome assessment, which may not capture delayed mesh-related complications that can manifest despite unremarkable early wound healing. To address this limitation, we have undertaken a longitudinal follow-up of the original cohort, with a particular focus on long-term outcomes in patients with poor preoperative glycemic control.

## Methods

Following approval from the institutional review board (IRB), adult patients with DM and preoperative HbA1c level ≥ 8.5% who underwent open, elective, clean (CDC wound class I) [8] ventral hernia repair (VHR) with concurrent TAR procedure and permanent synthetic mesh at the Cleveland Clinic Center for Abdominal Core Health (Cleveland, Ohio) between January 2014 to January 2024 were identified from the Abdominal Core Health Quality Collaborative (ACHQC). The ACHQC is a hernia-specific nationwide registry aimed at improving the quality of hernia care through patient-centered data collection, performance feedback to clinicians, and collaborative learning. Surgeons enter patient data prospectively in real-time during routine clinical care, including patient demographics, hernia characteristics, operative details, patient-reported outcomes (PROs), and postoperative follow-up information [9].

The present study builds upon our prior investigation evaluating short-term outcomes in 431 DM patients who underwent open, elective, clean VHR with concurrent TAR. To assess the long-term risk associated with poor preoperative GC, we identified a subset of patients with presurgical HbA1c ≥ 8.5% and followed them for a minimum of 12 months. HbA1c values were recorded at the most recent time point prior to surgery, within a maximum window of 90 days before the index procedure. Assessed outcomes included wound morbidity, mesh-related complications, overall postoperative morbidity, and hernia recurrence.

We evaluated wound morbidity at 30 days and a minimum of 12 months (± 4 weeks), including surgical site infections (SSI), surgical site occurrences (SSO), and surgical site occurrences requiring procedural intervention (SSOPI) [10]. SSIs were further categorized based on the CDC definition into superficial, deep, or organ space infections [11]. SSOs incorporated SSIs and other conditions such as wound cellulitis, non-healing incisional wounds, fascial disruption, skin or soft tissue ischemia, necrosis, serous or purulent wound drainage, stitch abscess, seroma, hematoma, infected or exposed mesh, or the development of an enterocutaneous fistula [10]. SSOPI incorporated any SSO that requires opening of the wound, wound debridement, excision of sutures, percutaneous drainage, or partial or complete mesh removal [10]). Additionally, 30-day and 12 months all morbidity including ileus, bowel obstruction, pulmonary embolism (PE), stroke, deep venous thromboembolism (DVT), myocardial infarction (MI), cardiac arrest, sepsis, septic shock, pneumonia, urinary tract infection (UTI), acute kidney injury (AKI), renal failure, re-intubation, and other complications were also reported.

Pragmatic hernia recurrence was determined based on prior work published by Krpata et al. [12]. In brief, clinical follow-up comprising surgeon evaluation and/or CT scans were employed to determine recurrence. If clinical and imaging evaluation did not occur, recurrence evaluation was carried out utilizing the Hernia Recurrence Inventory (HRI) [13]. In cases where a bulge was reported on HRI, the imaging could overrule that finding. If no other evaluation occurred, a bulge was considered a recurrence.

Clinical follow-up included surgeon evaluations at 30 days, 12 months, and 24 months, accompanied by CT scans at the 12 and 24-month follow-up points when available. Furthermore, we conducted a manual review of patients’ charts and images utilizing EPIC and Care Everywhere. Care Everywhere is Epic’s health information exchange software that allows sharing data with software of other participating medical centers. Patients who failed to have long-term clinical follow-ups were also tracked by direct phone calls to gather HRI, all potential complications mentioned above, readmissions, and reoperations since surgery.

### Statistical analysis

This study was designed as a descriptive longitudinal follow-up of a previously characterized cohort. No a priori sample size calculation was performed, and inferential testing across glycemic strata was not pre-specified, given the absence of contemporaneous control groups within this analysis. Continuous variables are presented as mean ± standard deviation or median (range), and categorical variables as counts and percentages with 95% Wilson confidence intervals for outcomes of interest. For each outcome, the denominator reflects the number of patients with documented assessment for that variable within the ACHQC registry; missingness was not imputed. Threshold analysis using ROC methodology and logistic spline regression was performed exploratorily across the observed HbA1c range to assess whether a discriminative cutoff could be identified within the poor glycemic control stratum, with the explicit caveat that the limited number of events constrains the precision of these estimates.

## Results

**Fig. 1:**
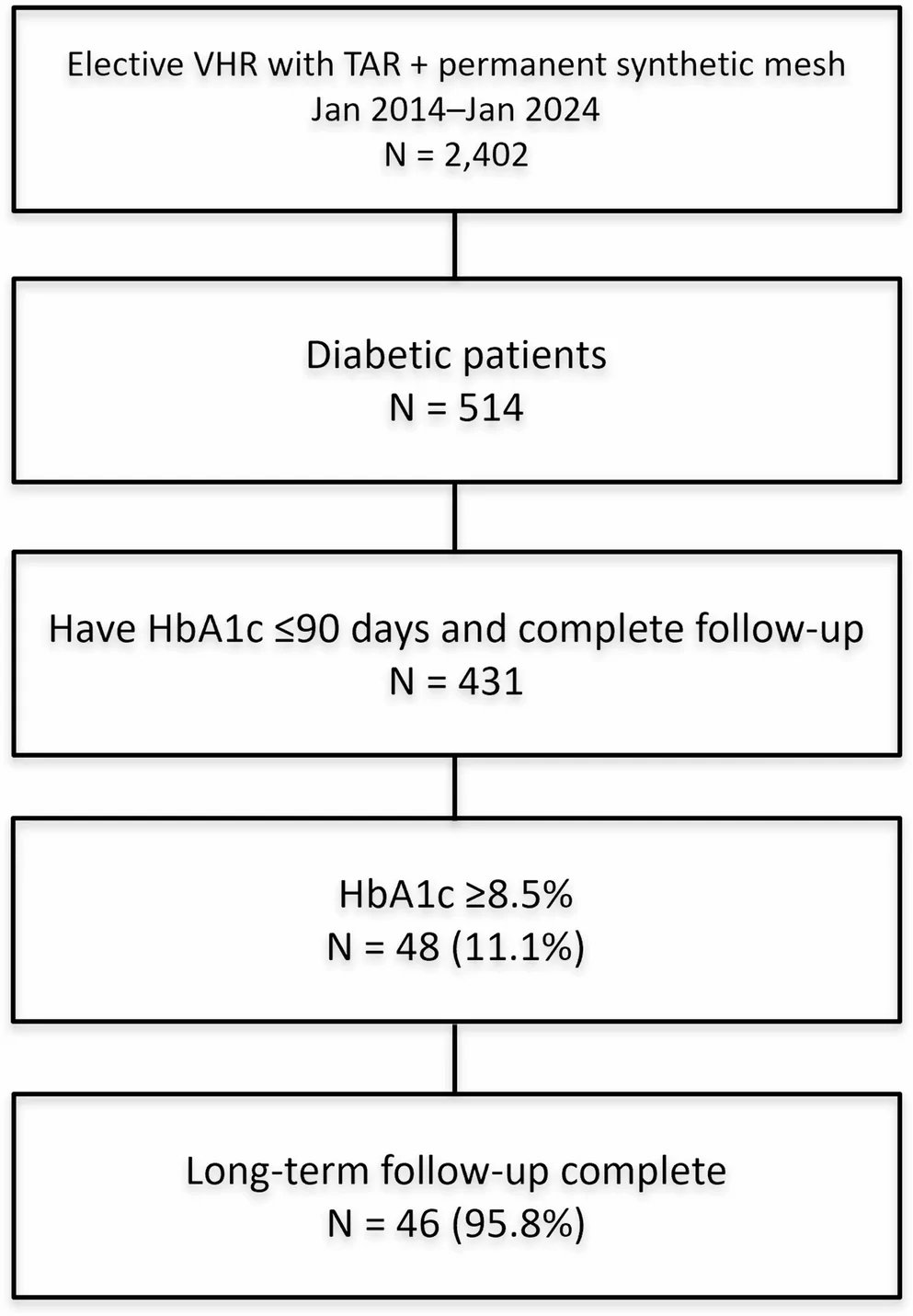
DM patients with HbA1c ≥ 8.5% undergoing open, elective AWR with TAR and permanent synthetic mesh between January 2014 to January 2024 at the Cleveland Clinic Center for Abdominal Core Health. Per-outcome denominators reflect documented ascertainment in the ACHQC registry: demographic, comorbidity, and operative variables *n* = 48; 30-day surgical site occurrence and seroma *n* = 40; 30-day SSI categories *n* = 39; systemic complications and length of stay *n* = 48; long-term outcomes (≥ 12 months) *n* = 46. Missingness was not imputed

During the study period (January 2014 to January 2024), 2,402 patients underwent open, elective, clean ventral hernia repair with TAR and permanent synthetic mesh at our institution. After excluding non-diabetic patients, 514 diabetic patients remained. We further excluded patients without preoperative HbA1c values within 90 days of surgery and those without complete follow-up data, leaving 431 patients. Among these, 48 patients (11.1%) were identified with poor preoperative glycemic control (HbA1c ≥ 8.5%). Of these, 46 (95.8%) completed long-term follow-up at a median of 22.3 months (range, 12–47 months). One patient died 14 months postoperatively from acute myocardial infarction without surgery-related complications, and one was lost to follow-up (Fig. 1).

**Fig. 2:**
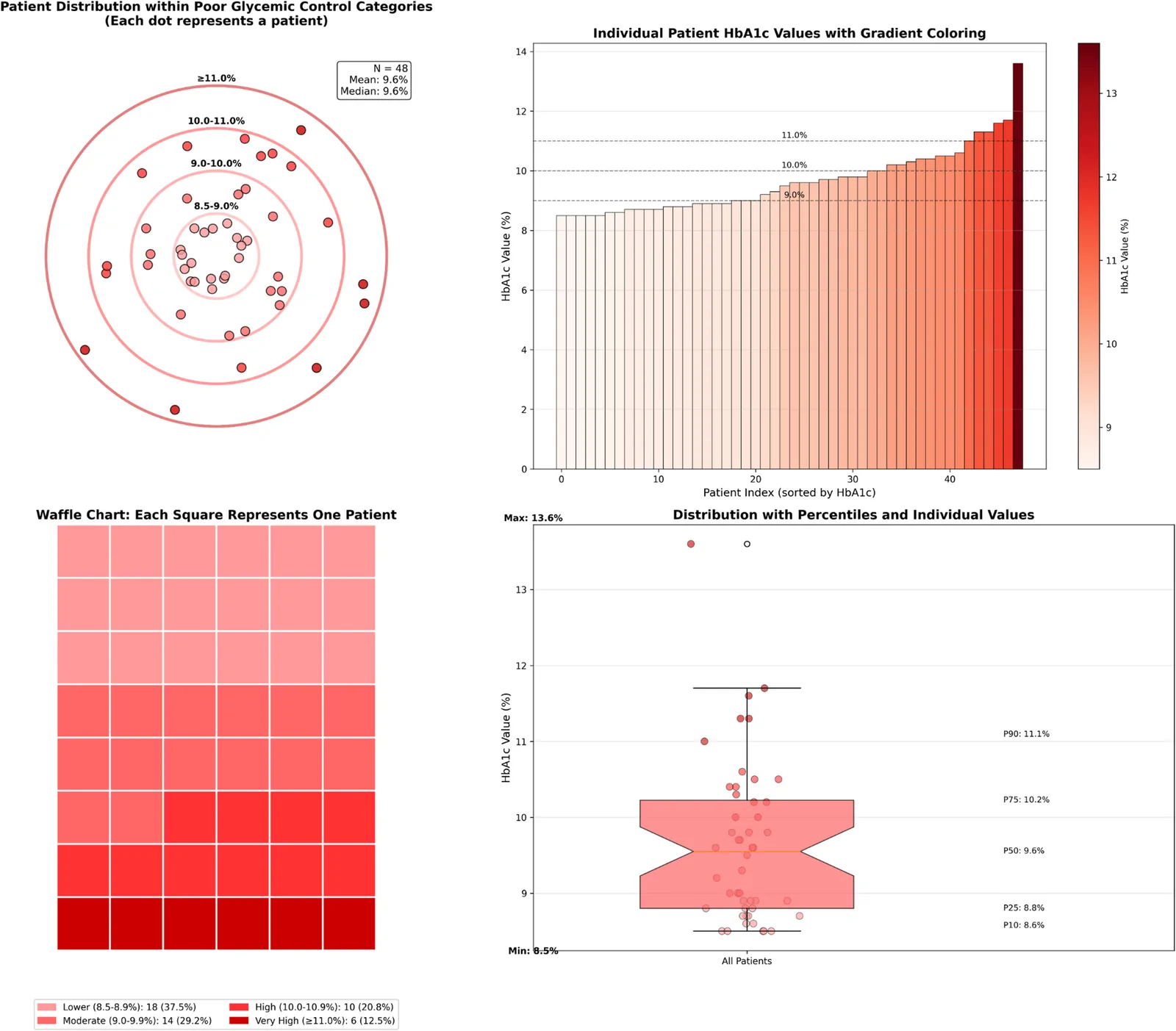
HbA1c distribution across the study population

The cohort included 19 female patients (39.6%), with a mean age of 58.9 ± 11.5 years and a mean BMI of 33.63 ± 5.2 kg/m². Common comorbidities included hypertension (*n* = 38, 79.2%), COPD (*n* = 7, 14.6%), and current tobacco use (*n* = 5, 10.4%). The majority were classified as ASA class III (*n* = 43, 89.6%) (Tables 1 and 2) (Fig. 2).

**Table 1:** Demographics and comorbidities characteristics

Demographics and comorbidities characteristics+A2:B23	Non-optimized-GC (HbA1c ≥ 8.5%) *N* = 48
Age, y (mean ± SD)	58.9 (11.5)
Female	19 (39.6%)
BMI (mean ± SD)	33.63 (5.2)
Hypertension	38 (79.2%)
COPD	7 (14.6%)
Liver Failure	0
Dialysis	0
Inflammatory bowel disease	1 (2.1%)
Crohn’s disease	0
Ulcerative colitis	1 (100%)
Immunosuppressants	4 (8.3%)
Anti platelet medications	6 (12.5%)
Anticoagulant medications	0
Current smoker	5 (10.4%)
ASA class	
2	4 (8.3%)
3	43 (89.6%)
4	1 (2.1%)
VHWG grade	
2	38 (79.2%)
3	10 (20.8%)

**Table 2:** Hernia and operative characteristics

Hernia and operative characteristics	Non-optimized-GC (HbA1c ≥ 8.5%) *N* = 48
Primary indication for surgery	
History bowel obstruction	5 (10.4%)
Enlarging Hernia	37 (77.1%)
Pain	47 (97.9%)
Hernia type	
Primary	0
Incisional	48 (100%)
Recurrent hernia	32 (66.7%)
Number of prior hernia repairs, mean (SD)	2.31 (1.4)
History of component separation	5 (10.4%)
History of open abdomen	1 (2.1%)
History of prosthetic mesh infection	6 (12.5%)
Previously infected mesh removed	5 (10.4%)
Hernia length, cm (mean ± SD)	22.19 (6.1)
Hernia width, cm (mean ± SD)	15.88 (6.7)
Mesh length, cm (mean ± SD)	38.46 (10)
Mesh width, cm (mean ± SD)	38.2 (10.3)
Fascial closure	45 (93.8%)
Fascial closure Suture	
Absorbable	45 (93.8%)
Permanent	0
Drain Placement: Retrorectus	48 (100%)
Retromuscular	48 (100%)
Subcutaneous	19 (39.6%)
Intraoperative complication	0
Operative time>2 h	44 (91.7%)

Five patients (10.4%) had a history of prior component separation, and six (12.5%) had a history of prosthetic mesh infection. All hernias were incisional, with 32 (66.7%) being recurrent and a mean of 2.31 ± 1.4 prior repairs. Mean hernia defect dimensions were 22.19 ± 6.1 cm in length and 15.88 ± 6.7 cm in width. Operative time exceeded 2 h in 44 patients (91.7%). Mean mesh dimensions were 38.46 ± 10.0 cm in length and 38.2 ± 10.3 cm in width. Fascial closure was achieved in 45 patients (93.8%) using absorbable suture. All patients received retromuscular drains; subcutaneous drains were placed in 19 patients (39.6%). No intraoperative complications were recorded (Table 2).

Postoperative 30-day and ≥ 12 months follow surgical site occurrence and overall morbidity are summarized in Table 3. A total of 13 SSOs (32.5%) were documented. SSI occurred in 5 patients (12.8%), including 1 case of superficial SSI (2.6%) and 4 cases of deep SSI (10.3%). Other SSOs included seroma (6 patients, 15.0%), cellulitis (1 patient, 2.6%), serous drainage (1 patient, 2.6%), hematoma (1 patient, 2.6%), and partially exposed mesh (1 patient, 2.6%). No cases of mesh infection or enterocutaneous fistula were observed. A total of 7 SSOPIs were documented at 30 days (17.5%, 95% CI 8.7–31.9%). At a minimum follow-up of 12 months, 5 SSOs (10.9%) were documented in 3 patients (6.5%, 95% CI 2.2–17.5%). Deep SSI was observed in 1 patient (2.2%), and 2 patients (4.3%) experienced non-healing incisional wounds. One patient (2.2%) developed mesh exposure, and another (2.2%) developed a mesh infection. A total of 2 SSOPIs were documented (4.3%, 95% CI 1.2–14.5%), comprising one percutaneous drainage (2.2%) and one partial mesh removal performed in the operating room (2.2%) (Table 3).

**Table 3:** Postoperative 30-day and ≥ 12 months follow surgical site occurrence and overall morbidity

Wound morbidity and clinical outcomes	30 days follow up	≥ 12 months follow up
Denominator (per outcome category, see footnote)	*N* = 48 / 40 / 39	*N* = 46
SSO	13/40 (32.5%, 95% CI 19.5–48.8%)	5/46 (10.9%, 95% CI 4.7–23.0%)
Superficial SSI	1/39 (2.6%, 95% CI 0.5–13.2%)	0
Deep SSI	4/39 (10.3%, 95% CI 4.1–23.6%)	1/46 (2.2%, 95% CI 0.4–11.3%)
Organ space SSI	0	0
Total SSI	5/39 (12.8%, 95% CI 5.6–26.7%)	1/46 (2.2%, 95% CI 0.4–11.3%)
Wound cellulitis	1/40 (2.5%, 95% CI 0.4–12.9%)	0
Wound purulent drainage	0	0
Stitch abscess	0	0
Contaminated mesh	0	0
Infected mesh	0	1/46 (2.2%, 95% CI 0.4–11.3%)
Seroma	6/40 (15.0%, 95% CI 7.1–29.1%)	0
Wound serous drainage	1/40 (2.5%, 95% CI 0.4–12.9%)	0
Hematoma	1/40 (2.5%, 95% CI 0.4–12.9%)	0
Non-healing incisional wound	0	2/46 (4.3%, 95% CI 1.2–14.5%)
Fascial disruption	0	0
Skin or soft tissue necrosis	0	0
Exposed mesh	1/40 (2.5%, 95% CI 0.4–12.9%)	1/46 (2.2%, 95% CI 0.4–11.3%)
Enterocutaneous fistula	0	0
SSOPI	7/40 (17.5%, 95% CI 8.7–31.9%)	2/46 (4.3%, 95% CI 1.2–14.5%)
Wound opening	5/39 (12.8%, 95% CI 5.6–26.7%)	0
Wound debridement	0	0
Percutaneous drainage	1/40 (2.5%, 95% CI 0.4–12.9%)	1/46 (2.2%, 95% CI 0.4–11.3%)
Partial mesh removal	2/40 (5.0%, 95% CI 1.4–16.5%)	1/46 (2.2%, 95% CI 0.4–11.3%)
Complete mesh removal	0	0
Reoperation	2/48 (4.2%, 95% CI 1.2–14.0%)	2/46 (4.3%, 95% CI 1.2–14.5%)
Hernia recurrence	0	4/46 (8.7%, 95% CI 3.4–20.3%)
Re-intubation	1/48 (2.1%, 95% CI 0.4–10.9%)	0
Post-Op bleeding requiring transfusion	1/48 (2.1%, 95% CI 0.4–10.9%)	0
Pneumonia	0	1/46 (2.2%, 95% CI 0.4–11.3%)
UTI	3/48 (6.3%, 95% CI 2.2–16.8%)	2/46 (4.3%, 95% CI 1.2–14.5%)
Septic shock	0	0
DVT	0	0
Pulmonary embolism	0	0
Stroke	0	0
Myocardial infarction	0	1/46 (2.2%, 95% CI 0.4–11.3%)
Cardiac arrest	0	0
Acute renal failure	0	1/46 (2.2%, 95% CI 0.4–11.3%)
Deceased at 30 days	0	-
Deceased at follow-up	-	1/46 (2.2%, 95% CI 0.4–11.3%)
Length of stay	5.92 (± 3.3)	-
Readmission	5/48 (10.4%, 95% CI 4.5–22.2%)	3/46 (6.5%, 95% CI 2.2–17.5%)

*SSO* = surgical site occurrence; *SSI* = surgical site infection; *SSOPI *= surgical site occurrence requiring procedural intervention; *UTI* = urinary tract infection; *DVT* = deep venous thrombosis. Categorical outcomes are presented as n/N (%, 95% Wilson confidence interval); continuous variables as mean ± SD. Denominators vary by outcome because of differing ascertainment windows in the ACHQC registry. Demographic, comorbidity, and operative variables are recorded for the full enrolled cohort (*n* = 48). At 30-day follow-up, surgical site occurrence and seroma assessment required documented wound evaluation (*n* = 40); SSI categorization required wound culture or organ-space documentation (*n* = 39); systemic complications and length of stay are reported on the full cohort (*n* = 48). Long-term outcomes (≥ 12 months) are reported on patients with completed extended follow-up (*n* = 46). Long-term procedural interventions (SSOPI) reflect events occurring beyond the 30-day window; one percutaneous drainage occurred at approximately 3 months and one partial mesh removal at approximately 4 years. Missingness was not imputed

Among the four patients who experienced long-term wound or mesh-related complications, the first was a 61-year-old male (HbA1c 10.0%, BMI 32.7) with a history of active smoking and a non-healing wound following prior colectomy, who presented with an 18 × 22 cm hernia. He underwent TAR with a 50 × 50 cm Prolene Soft Mesh. On postoperative day (POD) 12, he developed a deep SSI, managed with bedside wound opening, followed by complete wound healing. A recurrent hernia was first recognized within 12–18 months of surgery. Four years postoperatively, he developed a delayed mesh infection requiring partial mesh removal. The second patient was a 53-year-old female (HbA1c 9.6%, BMI 37.7) with a 20 × 36 cm recurrent hernia following four prior repairs, including anterior component separation. She underwent TAR with a 50 × 50 cm Prolene Soft Mesh and developed a wound infection on POD 10. Initial management included in-office wound opening, but subsequent operative debridement and partial mesh excision were required. She later experienced a hernia recurrence and underwent a successful repeat TAR procedure six years after the index operation. The third patient was a 55-year-old male (HbA1c 8.8%, BMI 47.4) with a 30 × 27 cm recurrent hernia and loss of domain. Following TAR with a 50 × 50 cm Prolene Soft Mesh, he remained intubated postoperatively due to elevated intra-abdominal pressure. A hematoma developed early in the postoperative course but required no immediate intervention, and the patient was discharged on POD 7. Three months later, the hematoma became infected and necessitated surgical drainage without mesh removal. A recurrent bulge was first recognized within 12–18 months of the index operation; cross-sectional imaging subsequently demonstrated a central mesh fracture, and he underwent laparoscopic intraperitoneal underlay mesh (IPUM) repair approximately three years postoperatively. The fourth patient was a 64-year-old female (HbA1c 9.2%, BMI 34.1) with a 24 × 17 cm recurrent incisional hernia following two prior repairs, presenting with chronic pain and progressive enlargement. She underwent TAR with a 50 × 50 cm Prolene Soft Mesh. On postoperative day 9, she developed a deep SSI managed initially with bedside wound opening; ongoing purulent drainage and an incompletely controlled wound bed prompted operative debridement and partial mesh excision during the index admission. The wound subsequently healed by secondary intention. Approximately 18 months postoperatively, she presented with a symptomatic bulge at the prior repair site, with recurrence confirmed on cross-sectional imaging. She was offered staged reoperation and is currently undergoing prehabilitation prior to repeat abdominal wall reconstruction.

Pragmatic hernia recurrence was observed in 4 of 46 patients (8.7%, 95% CI 3.4–20.3%) at a median follow-up of 22.3 months (range, 12–47 months). The median time to recurrence recognition was 15 months postoperatively (range, 12–18 months). All four recurrences were first recognized within 12–18 months of the index operation and occurred exclusively in patients who had experienced early wound morbidity (4/4); three of the four had also undergone partial mesh excision or removal (3/4). Later procedures at three and six years represent definitive recurrence repair rather than the time of first recurrence recognition. No recurrences were documented among patients with uncomplicated postoperative courses (Table 4).

**Table 4:** Long-term Hernia Recurrence in Patients with HbA1c ≥ 8.5%

Variable	HbA1c ≥ 8.5% (*N* = 46)
Median follow-up, months (range)	22.3 (12–47)
Patients with ≥ 24-month follow-up	28 (60.9%)
30-Day Follow-up	
Patient-reported recurrence	0 (0%)
Clinical recurrence	0 (0%)
Pragmatic recurrence	0 (0%)
≥ 12-Month Follow-up	
Patient-reported recurrence	1/46 (2.2%, 95% CI 0.4–11.3%)
Clinical recurrence	2/46 (4.3%, 95% CI 1.2–14.5%
Pragmatic recurrence	4/46 (8.7%, 95% CI 3.4–20.3%)
Recurrence Characteristics	
Median time to recurrence, months (range)	15 (12–18)
Recurrence in patients with mesh removal	3/4 (75.0%)
Recurrence in patients with early wound complications	4/4 (100%)

*HRI* = Hernia Recurrence Inventory; *CT *= computed tomography. Recurrence proportions are presented with 95% Wilson confidence intervals; denominators reflect the long-term-followed cohort (*n* = 46)

**Fig. 3:**
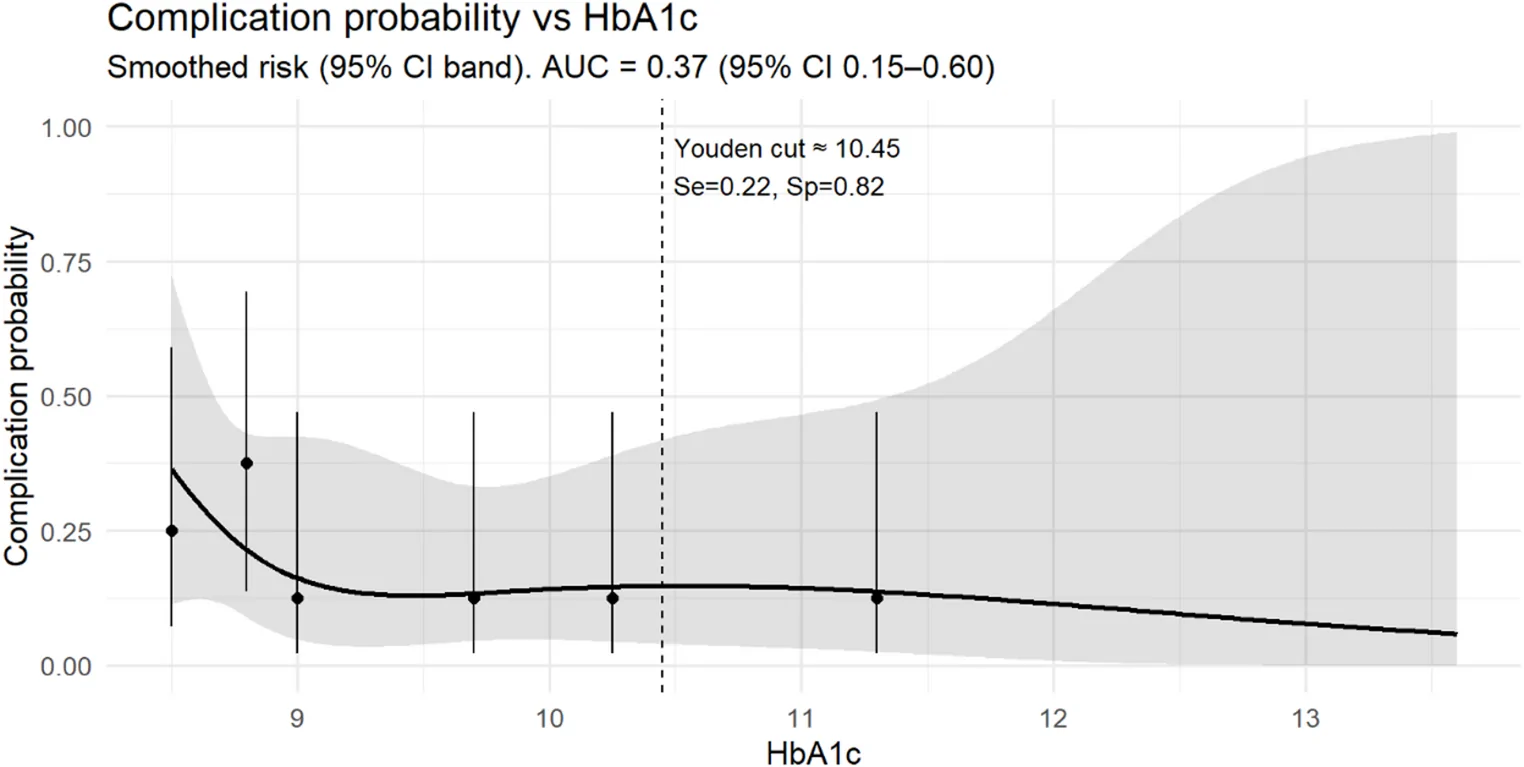
Relationship between preoperative HbA1c levels and postoperative complication probability

To evaluate whether a specific HbA1c threshold could predict postoperative complications, threshold analysis using ROC curve methodology and logistic spline regression was performed across the full range of observed HbA1c values (8.5–11.6%, mean 9.51 ± 0.86%). Logistic spline regression demonstrated a relatively flat predicted complication probability across the observed range, without a sharp inflection point. Youden’s J statistic identified an optimal statistical cutoff at approximately 10.45% HbA1c, yielding an AUC of 0.37 (95% CI: 0.15–0.60), sensitivity of 0.22, and specificity of 0.82 (Fig. 3).

## Discussion

This study represents a longitudinal extension of our prior investigation examining the impact of diabetes mellitus and preoperative glycemic control on short-term wound morbidity following open, elective, clean ventral hernia repair with TAR and permanent synthetic mesh [3]. In our original analysis, which encompassed 431 diabetic patients stratified by preoperative HbA1c, we observed that while diabetes mellitus was independently associated with increased wound morbidity relative to non-diabetic controls, no statistically significant differences in SSI, SSO, SSOPI, or mesh-related complications were detected across glycemic control strata at 30 days. These findings prompted the present analysis, which specifically tracks this high-risk subgroup longitudinally to determine whether the absence of early wound events accurately reflects the long-term clinical trajectory in poorly controlled diabetic patients undergoing complex AWR.

The central finding of the current study is that, at a median follow-up of 22.3 months, the majority of patients with poor preoperative glycemic control who experienced an uncomplicated early postoperative course remained free of long-term wound morbidity and hernia recurrence. Among the 46 patients who completed extended follow-up, four (8.7%) developed significant long-term wound or mesh-related complications necessitating procedural intervention. Critically, all four documented hernia recurrences occurred in patients who had experienced early wound morbidity (4/4); three of these four had also undergone partial mesh excision or removal (3/4). No recurrences were observed in patients with uncomplicated early wound healing. Within this cohort, recurrence segregated entirely with early wound morbidity rather than with HbA1c value or distribution, an internal observation that, while exploratory, suggests that the postoperative wound trajectory may carry stronger prognostic weight than the preoperative glycemic metric. The wide confidence intervals around these point estimates (e.g., 3.4–20.3% for pragmatic recurrence and 4.7–23.0% for long-term SSOPI) reflect the limited precision of estimates derived from this small cohort and do not exclude clinically meaningful risk; these values should be interpreted as exploratory. Importantly, we emphasize at the outset that this analysis lacks a contemporaneous internal comparator. Because no concurrent group of better-controlled diabetic patients managed under the same protocols was analyzed here, the present data cannot establish whether outcomes in this poorly controlled stratum differ from those of better-controlled diabetic patients treated at our institution under identical protocols. Comparison against external published series — which differ in case-mix, follow-up duration, and outcome definitions — provides context but does not substitute for such an internal comparison. The observations that follow should be read with this constraint in mind.

To contextualize these outcomes within the broader TAR literature, the observed long-term complication rates in the present cohort warrant direct comparison with published benchmarks. In the largest single-center long-term analysis of TAR outcomes to date, Zolin et al. reported a composite hernia recurrence rate of 10% at a median follow-up of one year in 1,203 patients undergoing elective, open TAR with permanent synthetic mesh [14]. The present study demonstrated a long-term SSOPI rate of 4.3%, a partial mesh removal rate of 2.2%, and a hernia recurrence rate of 8.7% at a median follow-up of 22 months. While lower than the 2-year composite recurrence of 21% reported by Zolin et al., this difference should be interpreted cautiously given the limited sample size and descriptive nature of our cohort. At the population level, the meta-analysis by Vasavada and Patel, encompassing 22 studies and 5,284 patients, reported pooled SSO, SSOPI, and recurrence rates of 21.7%, 9.8%, and 6%, respectively [15]. However, relatively short follow-up across included studies limits interpretation of recurrence. Similarly, the systematic review by Wegdam et al. reported a 15% SSO rate and 4% recurrence at 2 years across five centers, though incomplete follow-up and inconsistent recurrence assessment likely resulted in underestimation [16]. In a single-surgeon series by Turcotte et al. utilizing macroporous polypropylene mesh, 183 patients followed at a median of 24.7 months demonstrated overall complication, SSO, SSI, and recurrence rates of 16.4%, 10.4%, 3.8%, and 2.7%, respectively [17]. However, this is a lower-complexity cohort reflected by a median hernia width of 12 cm. Among series specifically evaluating TAR in previously recurrent hernias, a patient profile that mirrors our cohort, in which 66.7% had recurrent hernias, Han et al. reported a 5% recurrence rate at a mean follow-up of 15.5 months [18]. The 8.7% pragmatic recurrence proportion should be regarded as a preliminary estimate rather than a stable rate. All four recurrences were recognized within 12–18 months, and in a cohort this enriched for recurrent disease, the recurrence curve is unlikely to be fully mature at a median follow-up of 22.3 months. Additional events may emerge with longer observation, and the present figure may therefore underestimate the eventual cumulative recurrence.

In an effort to determine whether a specific HbA1c threshold within the poor glycemic control range might confer additional predictive value, we performed threshold analysis using ROC methodology and logistic spline regression across the full observed HbA1c range. The logistic spline demonstrated a flat predicted complication probability without a discernible inflection point, and the optimal Youden-derived cutoff of approximately 10.45% yielded an AUC of 0.37, indicating no better than chance. These results are consistent with our prior analysis and with the other studies suggesting that HbA1c, while a useful integrated marker of chronic glycemic burden, showed limited discriminative capacity within this small cohort for predicting postoperative wound events at the individual patient level [3, 7]. This finding should be interpreted as exploratory given the low event count and is not generalizable beyond the observed range. Of note, AUC values below 0.5 derived from a small number of events should be interpreted as consistent with the absence of a usable discriminative threshold rather than as evidence of an inverse association.

To date, the evidence evaluating the relationship between preoperative glycemic control and postoperative outcomes in hernia surgery remains limited and largely extrapolated from heterogeneous surgical populations. Much of the existing literature is derived from large administrative datasets encompassing diverse procedures and patient cohorts, limiting applicability to elective clean AWR [19–20]. In the retrospective study by Dronge et al., which utilized Veterans Affairs NSQIP data from a single VA healthcare system and included 490 diabetic patients who underwent major noncardiac surgery across diverse disciplines (including urological, gastrointestinal, vascular, orthopedic, thoracic, and neurological procedures), HbA1c > 7% was associated with an increased risk of postoperative infectious complications, with an adjusted odds ratio of 2.13 (95% CI 1.23–3.70) [19]. Similarly, Underwood et al. reported that HbA1c levels exceeding 8% correlated with prolonged hospital stays; however, their cohort included both emergent and non-emergent cases across varied surgical settings, further limiting the applicability of their findings to elective clean hernia surgery [20]. These datasets, while broadly applicable, do not specifically address the distinct wound biology and physiological demands of open AWR, wherein extensive abdominal wall dissection, creation of large retromuscular spaces, and implantation of wide synthetic meshes impose a wound healing challenge. Conversely, Acott et al., analyzing 38,989 NSQIP patients who underwent major surgical procedures across multiple specialties and institutions, found no detectable correlation between preoperative HbA1c levels and the risk of SSI, despite the large cohort size and the statistical power inherent to such a dataset [21]. This finding is supported by the systematic review of Rollins et al., which included 20 studies encompassing 19,514 diabetic patients from a range of surgical specialties published between 1980 and 2014 [22]. That review concluded that elevated preoperative HbA1c was not definitively associated with increased postoperative morbidity or mortality. Hernia-specific data remain particularly sparse. To our knowledge, Al-Mansour et al. are the only investigators, aside from our group, to directly evaluate the relationship between HbA1c and wound outcomes in a hernia cohort, demonstrating no significant association [7]. Importantly, their study included heterogeneous surgical techniques, including both open and minimally invasive approaches, multiple CDC wound classifications, varied hernia repair methods, and different mesh planes. The present study contributes to this body of evidence by examining long-term outcomes in the most challenging glycemic subgroup of a well-defined and operatively homogeneous cohort and by extending the follow-up horizon beyond the 30-day window that has historically constrained this literature.

While the present study cannot definitively exclude a role for preoperative glycemic status in determining postoperative outcomes, the cumulative evidence from our group is consistent with the hypothesis that, within this cohort and setting, preoperative HbA1c carries limited stand-alone discriminative value for wound morbidity and recurrence; this remains a cohort-specific, hypothesis-generating observation requiring confirmation in adequately powered comparative analyses. In our prior analysis, no significant association was identified between preoperative HbA1c levels and short-term wound morbidity across glycemic control strata [3]. The current study extends this observation longitudinally, demonstrating that long-term surgical site occurrences and recurrence rates in patients with poor preoperative glycemic control do not appear to exceed those reported in the broader TAR literature for comparably complex patient populations [23–24]. Taken together, these findings suggest that while diabetes mellitus as a diagnosis is associated with increased wound morbidity, the preoperative HbA1c level appeared less informative for individual risk stratification within this cohort. This remains a cohort-specific, hypothesis-generating observation that should not be generalized to the broader diabetic AWR population.

An important consideration in interpreting these findings is that, although overall complication and recurrence rates were comparable to those reported in the literature, the severity of complications in this cohort may be perceived as higher, given that poorly controlled diabetes is believed to predispose patients to more complex postoperative courses once complications occur [25]. While our study design does not permit direct comparative assessment of complication severity, the observed rates of clinically meaningful events, including deep or organ-space SSIs, mesh-related complications, and mesh excision, are consistent with those reported in contemporary TAR series.

From a practical standpoint, the findings from our prior study on short-term surgical site morbidity, together with the present analysis of long-term outcomes, have important implications for shared decision-making. Patients with diabetes should be counseled that AWR carries a substantial risk of wound morbidity and overall complications, and that optimization of glycemic control remains an important goal for overall health. However, in cases where optimal glycemic control cannot be achieved, our data suggests that proceeding with surgery may be reasonable. Many patients undergoing AWR present with large, symptomatic hernias that significantly restrict physical activity, impair core function, and negatively affect body image. These factors may, in turn, hinder weight control and glycemic optimization, creating a self-perpetuating cycle of declining metabolic and functional health. Accordingly, rigid reliance on HbA1c thresholds alone may unnecessarily delay or preclude intervention, potentially perpetuating functional impairment and limiting the patient’s ability to engage in health-promoting behaviors. A further consideration concerns the perioperative period itself. Because postoperative glucose management data were not systematically captured, we cannot distinguish between two explanations for the limited prognostic signal observed: that preoperative HbA1c is inherently weakly discriminative in this population, or that standardized perioperative optimization at a high-volume specialty center attenuates a signal that might otherwise be apparent. These possibilities carry materially different implications: if the latter holds, the reassurance offered by these data may not transfer to lower-volume or less protocol-driven settings, where the prognostic weight of preoperative HbA1c could be greater. This reinforces our position that the findings are setting-specific and should not be used to justify forgoing glycemic optimization, particularly outside high-volume centers.

This study has several important limitations. First, although data were collected prospectively through the ACHQC, the analysis itself is retrospective in nature and subject to the inherent limitations of observational study design. Second, the cohort is small, with only 46 patients completing long-term follow-up, precluding formal inferential statistical comparisons and limiting the study to a descriptive analysis. However, our study focuses on a specific and highly morbid operative scenario involving large incisional hernias undergoing complex open reconstruction with TAR, which lends clinical significance to even a modest sample size. The absence of a concurrent comparison group, such as diabetic patients with optimal or sub-optimal glycemic control followed over the same period, limits our ability to draw conclusions about the relative risk attributable to poor glycemic control specifically. A longitudinal follow-up of diabetic patients across all glycemic control strata is currently ongoing at our institution, and findings will be reported once follow-up is sufficient. Third, while we attempted to stratify outcomes by HbA1c level within the poor glycemic control range, the majority of patients in this cohort had HbA1c values between 8.5% and 10%, and caution is therefore advised in extrapolating our findings to patients with more markedly elevated levels. It is also important to note that this cohort comprised exclusively elective, clean cases, and our findings may not be applicable to more complex surgical scenarios, including urgent presentations or contaminated operative fields. Additionally, postoperative glucose management data were not systematically captured, as discussed above. As diabetes management continues to evolve, the potential impact of newer pharmacological agents and treatment strategies on surgical outcomes warrants evaluation in future studies. Finally, this study represents a single-center experience performed by high-volume abdominal wall reconstruction surgeons, and the findings may not be directly generalizable to other institutions or operative settings. We therefore encourage surgeons to monitor their own outcomes, critically assess the applicability of these findings to their practice, and incorporate this information into shared decision-making conversations with their patients.

In this descriptive long-term follow-up, preoperative HbA1c did not visibly stratify long-term surgical risk within the poorly controlled stratum of a single-center diabetic cohort managed with standardized perioperative optimization at a high-volume center. All observed recurrences occurred in the context of early wound morbidity, with none observed among patients with uncomplicated postoperative courses. These findings are hypothesis-generating and should not be interpreted as licensing surgery in poorly controlled diabetic patients without individualized risk assessment, particularly outside high-volume specialty centers. Whether the apparent limited stand-alone discriminative value of preoperative HbA1c reflects a true biological signal or the protective effect of perioperative optimization and surgical expertise will require dedicated comparative analyses. Pending such confirmation, we suggest that rigid HbA1c thresholds be interpreted within a broader framework of individualized risk assessment in shared decision-making for symptomatic patients with significant functional impairment.

## Data Availability

Data supporting the findings of this study are available from the corresponding author upon reasonable request, subject to institutional and registry data governance policies.
